# A New Grand Challenge in Rhinology: An Intranasal COVID Vaccine

**DOI:** 10.3389/falgy.2022.881118

**Published:** 2022-05-20

**Authors:** Glenis Kathleen Scadding

**Affiliations:** ^1^University College London Hospitals NHS Foundation Trust, London, United Kingdom; ^2^Division of Immunity and Infection, University College London, London, United Kingdom

**Keywords:** SARS-CoV-2, COVID-19, systemic vaccines, intranasal vaccine, IgA

The decision to give systemic COVID vaccines to children aged 5–11 is finely balanced, since very few young children suffer severely with SARS-CoV-2 infection, probably because of their more effective innate immunity (1). In addition recent data suggests that Pfizer vaccine efficacy is low in 5–11 year olds (2). Presumably the need to reduce viral transmission and hence the development of new strains is one consideration. Some 12 months after the first COVID-19 vaccine received WHO Emergency Use Listing (EUL), more than 9 billion COVID-19 vaccine doses have been administered globally. These systemic vaccines have been remarkably successful in reducing morbidity and mortality from SARS-CoV-2 but have only modest effect on viral transmission (3), probably because systemic vaccination does not provide sufficient mucosal protection (4).

If there is a need to vaccinate children worldwide then an alternative mucosal route might be safer, simpler and superior in reducing transmission, as well as more acceptable to children and their carers.

SARS-CoV-2 enters the body mainly *via* the ciliated cells in the upper airway (5). The nose defends the lower airways and the lungs and provides a route for therapy (6). Part of this defence is innate, involving muco-ciliary clearance, interferon, nitric oxide gas; the adaptive (educable) immune system is also involved. This is the mucosal, not the systemic immune system. The major relevant antibody is not IgG, but IgA. Local generation of secretory IgA (SIgA) which constitutes the body's biggest humoral immune system can exclude pathogens, neutralize viruses inside virus-infected epithelial cells and can redirect antigens in the lamina propria to the lumen (7). Viral upper airway infections such as influenza, rhinovirus and SARS-CoV-2 are associated with an increase of S-IgA in nasal lavage. IgA plays an important role in the protection against influenza in humans (8, 9). Mice lacking S-IgA have increased viral load after intranasal challenges (10) and transfer of nasal IgA from immunized to naïve mice leads to protection (11). Volunteers infected with coronavirus 229E had IgA antibody in nasal fluids associated with reduced periods of viral shedding (12). Elite athletes with increased viral colds show decreased salivary S-IgA (13–15). In COVID infection IgA antibodies against SARS-CoV-2 were elevated in nasal fluids, tears, and saliva (16, 17). Salivary antibodies persisted for at least 3 months (18).

Systemic (intramuscular) immunization does not confer significant mucosal immunity (4). The reverse is not true. The advantages of the intranasal route, in addition to rapidity and needle-free administration, include the generation of both mucosal (SIgA) and circulating (IgG and IgA) antibodies, as well as T cell responses. Intranasal vaccination induces resident memory T cells (T_RM_) which provide stronger protective immunity than circulating T cells (19) and could be particularly beneficial for rapidly mutating pathogens, such as SARS-CoV-2, where antibody-mediated protection is swiftly evaded (20). Intranasal vaccination might achieve desirable results, such as reduced viral transmission, not obtained with systemic immunization. It is also less likely to result in systemic inflammatory problems such as pericarditis and myocarditis, seen in systemically-immunized adolescents. Adverse events of vaccination such as vaccine-associated enhanced respiratory (VAERD) disease (21), as seen in some newly-infected children who have received systemic inactivated measles or RSV immunization, is less likely with a nasal vaccine which should result in inability of the virus to combine with its receptor and immune exclusion by phagocytosis after combination with divalent IgA, linked by secretory piece (6), thus obviating lower respiratory tract infection. Th2 stimulation, seen with COVID infection and with current systemic COVID vaccines, might be avoided by use of a suitable adjuvant (22).

Intranasal vaccines are already available against influenza, others are under development against COVID 19 (23). Used in UK children live attenuated nasal influenza vaccine shows consistently good effectiveness and indirect protection extending to both older and younger age groups has been demonstrated (24). Another advantage of intranasal influenza vaccine over the injection route is the induction of cross-reactive antibodies which provide variant strain protection (25). This concept may also apply to SARS-CoV-2, though as yet there is no evidence. The nasally applied influenza virus has been temperature-adapted so that it can only replicate in an environment as cold as the nose, not in the warmer lung. The Omicron variant of SARS-CoV-2 also appears to be similarly restricted, causing significantly less lung disease than its predecessors, whilst improving immunity against the more pathogenic delta variant (26). Site—directed mutagenesis might provide a similar asymptomatic or minimally symptomatic variant confined to the nose and appropriate as a vaccine.

In order to evoke a nasal mucosal immune response the SARS-CoV-2 virus would need to evade the normal nasal defence mechanisms such as mucociliary clearance, and achieve absorption through the mucosa in order to reach the local nasal associated lymphoid tissue (NALT). The spike protein should enable viral adhesion *via* its affinity for ACE 2 and TLR4 receptors which are present on the nasal epithelium (27). Children have lower respiratory ACE 2R expression than adults, topical corticosteroids also reduce ACE 2R expression. If the virus fails to interact with the nasal mucosa it will be moved by muco-ciliary clearance to the throat and swallowed, reaching the gut. Here a mucosal response can also be initiated by the local associated lymphoid tissue (GALT), which as part of the mucosal associated lymphoid tissue (MALT), can protect the respiratory tract (28). In COVID-19 infection (GI) symptoms predict better clinical outcomes with significantly lower death rates (29).

If nasal immunization is insufficient to provide protective immunity, then an alternative strategy would be to initiate a response by systemic vaccination, but to boost this nasally (30).

Animal studies suggest the feasibility of a nasal approach. A Newcastle disease virus (NDV)-based SARS-CoV-2 vaccine encoding a human codon-optimized full-length wild-type spike (S) protein of SARS-CoV-2 (rNDV-S) *via* a reverse genetic approach (31) and given as two intranasal doses to mice resulted in systemic humoral and cell-mediated immune responses with high levels of SARS-CoV-2 NAbs and anti-SARS-CoV-2 immunoglobulin A (IgA) and IgG2a. Similarly, hamsters, nasally vaccinated then challenged with SARS-CoV-2, were protected against lung infection and inflammation with reduced viral shedding into nasal turbinate and lungs. Intranasal immunization of rNDV-S has the potential to control SARS-CoV-2 infection at the site of inoculation, preventing both disease and transmission (32). A single intranasal spray of a cold-adapted live-attenuated COVID-19 vaccine induced potent humoral, cellular, and mucosal IgA immune responses in human-ACE2 transgenic mice who were completely protected from viral challenge without detectable virus in nasal turbinates and vital organs (33). In rhesus macaques, a single intranasal dose of adenovirus-vectored vaccine protects against upper and lower SARS-CoV-2 respiratory infection (34). A state-of-the-art summary of intranasal COVID-19 vaccines in development is recently available including the few in clinical trials (35). An *ex-vivo* model of the human nose might facilitate development and assessment of putative vaccines (36). When considering the next generation of COVID and other respiratory vaccines the intranasal route should not be completely ignored, as it is in a recent publication (37).

## Author Contributions

The author confirms being the sole contributor of this work and has approved it for publication.

## Conflict of Interest

GS was the (unpaid) Chair of the Data Monitoring Committee for the SNIFFLE Trials of influenza vaccination in egg allergic children.

## Publisher's Note

All claims expressed in this article are solely those of the authors and do not necessarily represent those of their affiliated organizations, or those of the publisher, the editors and the reviewers. Any product that may be evaluated in this article, or claim that may be made by its manufacturer, is not guaranteed or endorsed by the publisher.
